# Human Immunodeficiency Virus and Heparan Sulfate: From Attachment to Entry Inhibition

**DOI:** 10.3389/fimmu.2013.00385

**Published:** 2013-11-20

**Authors:** Bridgette J. Connell, Hugues Lortat-Jacob

**Affiliations:** ^1^University of Grenoble Alpes, Institut de Biologie Structurale, Grenoble, France; ^2^Centre National de la Recherche Scientifique, Institut de Biologie Structurale, Grenoble, France; ^3^Commissariat à l’Énergie Atomique, Direction des Sciences du Vivant, Institut de Biologie Structurale, Grenoble, France

**Keywords:** heparan sulfate, glycosaminoglycan, CCR5/CXCR4, gp120, V3 loop, co-receptor binding site, HIV-1, attachment and entry inhibition

## Abstract

By targeting cells that provide protection against infection, HIV-1 causes acquired immunodeficiency syndrome. Infection starts when gp120, the viral envelope glycoprotein, binds to CD4 and to a chemokine receptor usually CCR5 or CXCR4. As many microorganisms, HIV-1 also interacts with heparan sulfate (HS), a complex group of cell surface associated anionic polysaccharides. It has been thought that this binding, occurring at a step prior to CD4 recognition, increases infectivity by pre-concentrating the virion particles at the cell surface. Early work, dating from before the identification of CCR5 and CXCR4, showed that a variety of HS mimetics bind to the gp120 V3 loop through electrostatic interactions, compete with cell surface associated HS to bind the virus and consequently, neutralize the infectivity of a number of T-cell line-adapted HIV-1 strains. However, progress made to better understand HIV-1 attachment and entry, coupled with the recent identification of additional gp120 regions mediating HS recognition, have considerably modified this view. Firstly, the V3 loop from CXCR4-using viruses is much more positively charged compared to those using CCR5. HS inhibition of cell attachment is thus restricted to CXCR4-using viruses (such as T-cell line-adapted HIV-1). Secondly, studies aiming at characterizing the gp120/HS complex revealed that HS binding was far more complex than previously thought: in addition to the V3 loop of CXCR4 tropic gp120, HS interacts with several other cryptic areas of the protein, which can be induced upon CD4 binding, and are conserved amongst CCR5 and CXCR4 viruses. In view of these data, this review will detail the present knowledge on HS binding to HIV-1, with regards to attachment and entry processes. It will discuss the perspective of targeting the gp120 co-receptor binding site with HS mimetic compounds, a strategy that recently gave rise to entry inhibitors that work in the low nanomolar range, independently of co-receptor usage.

## Introduction

HIV-1 is the causative agent of acquired immunodeficiency syndrome (AIDS), a condition in humans in which progressive failure of the immune system leads to the development of severe opportunistic infections and unusual malignant disorders (1). Infection occurs through the transfer of blood, semen, vaginal fluid, or breast milk, in which HIV-1 can be present as both free virus particles and/or within infected cells. The virus infects vital cells of the immune system, including CD4^+^ T-helper lymphocytes, macrophages, and dendritic cells, all of which are key to the development and orchestration of the immune response (2). This results in the targeted depletion of CD4^+^ T cells, the main function of which is to promote cytotoxic T-lymphocyte dependant killing of cells expressing foreign antigens and up regulate antibody production by B-lymphocytes. When CD4^+^ T cells numbers decline below a critical level (400/μl of blood) cell-mediated immunity is compromised and the body becomes progressively more susceptible to opportunistic infections (3). The first step of the HIV-1 replication cycle, attachment and entry into host cells, occurs through specific interactions between gp120, the glycoprotein which constitutes the surface unit of HIV-1 envelope spikes (Env) and the primary cellular receptor, CD4. This promotes further contacts between gp120 and members of the chemokine receptor family, among which CCR5 and CXCR4 are the most physiologically relevant and ultimately lead to the fusion of the viral and the host cell membranes (4). HIV-1 preferentially uses CCR5 during the acute phase of infection but, later in the course of HIV-1 infection progressing to AIDS, HIV-1 variants frequently appear that become adapted to utilize CXCR4.

Before encountering permissive CD4^+^ cells the virus may interact with several other alternative receptors, often referred as “attachment receptors” (5) such as Galactoside Ceramide (GalCer), present at the surface of epithelial cells, Mannose-Binding Lectin (MBL), Dendritic Cell Specific ICAM-3-Grabbing Non-integrin (DC-SIGN), or Heparan Sulfate Proteoglycans (HSPGs), the latter being present at the surface of virtually all cell types. Although these interactions generally do not permit infection *per se*, they can importantly affect mucosal cells (the portal through which HIV-1 enters in the body) attachment and transport across epithelial layers, tropism, tissue invasion, or cellular entry (6, 7). This review will discuss some aspects of the HIV-1-HSPG interaction and will describe how the biochemical characterization of this interaction led to the engineering of a new class of potential attachment and entry inhibitors.

## Heparan Sulfate Proteoglycans

Heparan sulfate proteoglycans are glycoproteins carrying one or more covalently bound heparan sulfate (HS) chains, a large anionic polysaccharide of the glycosaminoglycan (GAG) family, characterized by astonishing structural diversity and interactive properties. These complex molecules are widely distributed within tissues, and can be found at the cell surface such as the syndecans and glypicans, within the extracellular matrix such as agrin, perlecan, or type XVIII collagen, or intracellularly such as serglycin (8). Being predominantly and ubiquitously present in the extracellular milieu, these macromolecules are unsurprisingly playing essential roles in a vast number of biological processes occurring at the cell–cell and cell–matrix interface.

**Figure 1:**
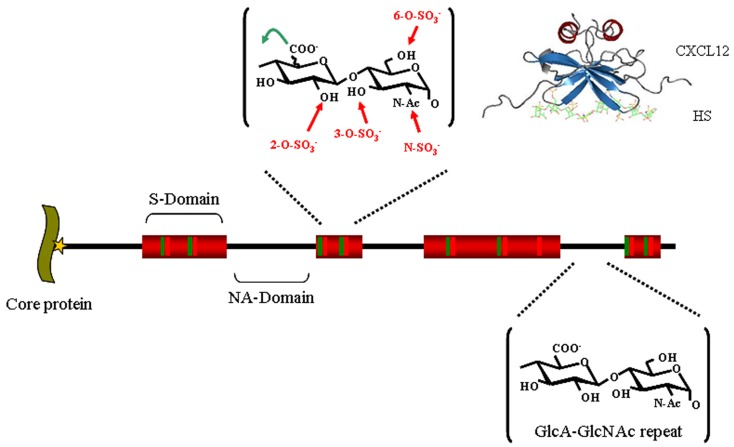
**Heparan sulfate structure**. HS, whose biosynthesis is initiated by the attachment of xylose (star) to specific serine residues in HSPG core proteins, followed by the formation of a linking tetrasaccharide (xylose-galactose-galactose-glucuronic acid), is initially polymerized by an enzyme complex composed of Ext1 and Ext2 as a GlcA-GlcNAc repeat (black). In restricted regions, called S-domains (shown in red), the chain is extensively modified by a series of enzymatic reactions that remove the acetyl group from GlcNAc residues and substitute the resulting free amino groups with sulfates, epimerizes the adjacent GlcA into L-iduronic acid (IdoA) and adds sulfates on various positions: the C2 of the IdoA (and less frequently that of the GlcA), the C6 of the GlcNS (and less frequently that of the GlcNac), and finally at the C3 of GlcNS or GlcN units. Altogether, these modifications can generate (the theoretical number of) 48 different disaccharides, whose combination within the S-domain gives rise to a large diversity of structures and make up binding sites for protein ligands, as depicted for example with a model of a CXCL12-HS complex [from Ref. (21)].

Over the past two decades, HSPGs have been found indeed to bind to a multitude of protein ligands, including cytokines, chemokines, morphogens, growth factors, adhesion and matrix molecules, receptors, enzymes, plasma proteins, etc. (8, 9). These interactions, which usually involve the HS chains, serve a large number of purposes. Functionally, HS has been known to affect the local concentration, the compartmentalization, the stability, the structure, and/or the activity of its ligands. Protein-HS interactions thus play critical roles, for example, in mediating the formation of chemokine gradients along which cells can migrate directionally (10–12), in providing a scaffold onto which two adjacent proteins such as growth factor-receptor complexes can interact (13), in protecting cytokines against proteolysis (14), in inducing protein conformational changes (15), in controlling or in restricting the diffusion of its ligands (16–18) thereby generating a local concentration of a given protein. As it will be described below, many microbial pathogens hijack HSPGs and take advantage of their interactive properties for their adhesion to host tissues and invasion of host cells. From a structural view point HSPGs’ multiple binding activities are believed to be closely related to the extended structural variability of the HS chain. It is a long (20–150 nm) and linear polysaccharide made of a repeating units of a 1 → 4 linked disaccharide motifs, comprising a glucuronic acid (GlcA) or its C-5 epimer, an iduronic acid (IdoA), and a *N*-acetyl- or *N*-sulfated-glucosamine (GlcNac or GlcNS), either or both of which may be *O*-sulfated at different positions. Variation in length, sulfation, and glucuronate/iduronate ratio, which occur in restricted domains of usually three to six disaccharides along the chain (Figure 1), generates a very large polydispersity and, as such, provides distinct docking sites for the various ligands of the polysaccharide (19, 20).

## HSPG and Pathogens

Attachment to host tissues is a critical step for most pathogens’ invasion and dissemination. It is therefore not very remarkable that due to its wide expression and large interactive properties, HS is used by many pathogens for that purpose (22–26). These include parasites, for example *Plasmodium falciparum* (27) bacteria, such as *Pseudomonas aeruginosa* (28), *Borrelia burdorferi* (29), or *Mycobacterium tuberculosis* (30), and many viruses, amongst which are found B (31), C (32), delta (33), and E (34) hepatitis viruses, Human Papillomavirus (35), Herpes viruses (36), HTLV-1 (37), or HIV-1 (38). Several lines of evidence have identified HS as an initial receptor for viral infection. Firstly, many capsid or envelope viral proteins bind to HS, secondly, elimination of cell surface HS is usually associated with increased cell resistance to infection that results from a reduction in the virus’ ability to bind to the cell surface (39), thirdly, soluble HS or HS like molecules, including heparin, a chemically related GAG and dextran sulfate, inhibit viral attachment and subsequent entry in cell culture experiments (40–42). Finally, it has been described that a number of viruses undergo cell culture adaptation changes resulting in an increased binding to HS (43–46). Together this suggests that selection during cell culture of mutants that bind HS with high affinity confer a selective advantage to the viruses. It has thus been thought that HS could facilitate concentration of the viral particles at the cell surface, restricting their diffusion to the quasi-two-dimensional network of polysaccharides around the cell and as such enhances the probability of access to specific entry receptors. HS can also capture viral particles at the surface of non-permissive cells, and then mediate *in trans* infection by presenting these viruses to attachment and entry receptors on permissive cells (47). HS binding can also go well beyond the simple attachment mechanism and can play a more direct role in cellular entry. This has been demonstrated in particular for HSV, a virus whose entry into epithelial cells involves several glycoproteins of the envelope (48). While the viral envelope glycoproteins gB and gC participate in the initial cell attachment through binding to HS, the gD, which binds to herpes virus entry mediator (HVEM) or to nectin, triggers fusion between host and viral membranes, but can also promote viral entry by interacting with a specific HS motif comprising a 3-*O*-sulfated glucosamine residue (49). Interestingly, a 3-*O*-sulfonated HS octasaccharide, produced by chemical means, was shown to inhibit the HSV-1 host-cell interaction (50), suggesting the use of HS derived molecules as therapeutic tools against viral pathogens.

## Where Does HIV-1 Meet HS?

HIV-1 is transmitted by viral exposure at the mucosal surfaces, which can occur in the genital tract (semen, blood) the intestinal tract (semen, blood, breast milk), or through the placenta (maternal blood), or the bloodstream (blood products). Within the context of vaginal or rectal transmission, HIV-1 must first cross a normally protective mucosal epithelium to reach the underlying dendritic cells, macrophages, and T cells which all express the virus primary receptor, CD4 and at least one of its two co-receptors, CCR5 or CXCR4, and are thus the three major cellular targets of HIV-1. In these early events of transmission, before specific cell infection, HS has been shown to play important roles in viral adsorption and dissemination. In the semen first, which is the main vector for HIV-1 dissemination, and which contains both free virions and infected leukocytes, it has been found that spermatozoa can capture HIV-1 in a HS dependant manner. Such spermatozoa-attached viruses are efficiently transmitted to dendritic cells, macrophages, and T cells (51) to which access could be made possible through mucosal microabrasions or through dendritic cell projections that extend to the luminal surface of the mucosa.

The mechanisms used by free or cell associated virions to cross an otherwise healthy mucosal barrier are not well known and might differ depending on the tissue sites (for example vaginal or rectal epithelium) where infection occurs (52). HIV-1 can interact with the epithelial cells and can traverse the epithelium through transcytosis, endocytosis followed by exocytosis or by penetrating the gaps in between cells, gaining access to susceptible leukocytes that will further propagate and spread the infection (53, 54). In this context, it has been well known that epithelial cells express large amount of HSPGs which can sequester HIV particles. For example, attachment of HIV to an ectocervical epithelium-derived cell line can be inhibited both by heparinase (an enzyme that depolymerizes HS and removes it from the cell surface) or by soluble heparin. Interestingly, it has been found that cell surface bound HIV particles remain infectious for at least 6 days, and upon co-culture with CD4^+^ cells, can be efficiently transmitted to its target cells (55). It has been also reported that HSPG significantly contributes to both attachment to the apical pole of – and transcytosis through – an endometrial epithelium-derived cell line (7). Similarly, cell-free HIV particles have been shown to transcytose (although with low efficiency) through primary genital epithelial cells, a process that was dependant on syndecan, one of the major HSPGs expressed by epithelial cells (56).

Finally, HS was recently found to be indispensable for gp120-mediated induction of TLR signaling in intestinal and genital epithelial cells. In the gp120-TLR-HS complex, HS was critical to activate the intracellular NF-kB pathway which lead to downstream synthesis of proinflammatory cytokines and chemokines, and whose upregulation was associated with tight junction disruption and loss of barrier function (57). Therefore, beyond acting as an ancillary attachment receptor, HS can contribute to barrier loss and initiation of immune activation that could be the first step in the characteristic chronic immune activation of HIV-1 pathogenesis.

Primary infection can also develop from viral exposure in the oral cavity (mother to infant nursing or during oral intercourse), where the palatine tonsil is a replication site for HIV-1. Studies aiming at characterizing the expression pattern for HIV-1 ligands on human palatine tonsils have shown that HS was largely present on both the surface of the stratified squamous epithelium and on the reticulated epithelium lining the tonsillar crypts and where it is likely to provide stable binding for the virus, allowing it to penetrate beneath the luminal surface and encounter CD4^+^ cells (58). Finally, it has also been shown that HIV-1 infection of trophoblasts is independent of CD4 but, at least partly, relies on HSPG. Mother to child vertical transmission of the virus is a major cause of HIV-1 infection in infants, and direct infection of trophoblasts, the cells that form the placental barrier, may cause this transmission (59).

HIV-1 is also trapped very efficiently by endothelial cells, which usually express large amounts of HSPG. Syndecan-3, for example, which delineates the contour of endothelial cells in lymphoid tissue high endothelial venules, does not substitute for HIV-1 entry receptors but captures HIV-1 and presents it to passing permissive T cells, thus mediating an *in trans* mechanism of infection. Furthermore, whereas unbound viruses lose infectivity in 1 day, syndecan-attached HIV-1 remains infectious for up to 1 week (60).

Finally, HSPGs also significantly contributes to HIV-1 invasion in the brain and neurological complications that often characterize AIDS patients. Whereas HIV-1 can enter the central nervous system within infected CD4^+^T-cell and monocytes that traffic across the blood brain barrier, several *in vitro* and *in vivo* reports described that free HIV-1 can be taken up by brain endothelial cells in a HS dependant manner, internalized and exocytosed, as a way to cross the blood brain barrier before infection and replication can occur in central nervous system cells such as microglia and astrocytes (61–63).

Although this review is devoted to HIV-1 attachment and entry, it is worth noting that, in addition to gp120, several other HIV proteins regulating various aspects of the virus life cycle also appeared to bind cellular HS after being released from HIV-infected cells. These protein-HS interactions contribute to trigger a variety of biological effects related to AIDS-associated pathologies. This includes p17, the matrix protein (64) which up-regulates cytokine production thus deregulating the functions of many immune cells; TAT, the transacting activator of transcription (65) which exerts angiogenic, cell proliferation, chemoinvasion activities and induces peripheral neuropathies, immune suppression, and tumorigenesis; and finally Vpr, the Viral protein R (66), which induces cell cycle arrest and apoptosis.

**Figure 2:**
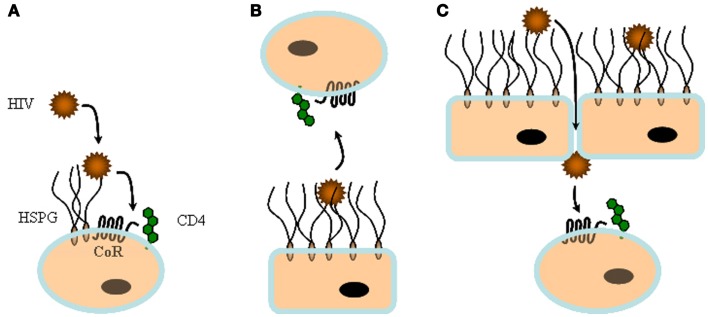
***In cis* and *in trans* capture of HIV-1 by heparan sulfate**. HS can play multiple roles during viral infection. **(A)** On top of cells that express large amount of HS, but low CD4, such as macrophages, HS can capture viral particles and facilitate *in cis* subsequent interaction with specific entry receptors. **(B)** HS from non-permissive cells such as endothelia or epithelia can sequester HIV-1 and then mediate *in trans* infection by presenting the virus to permissive cells. **(C)** HS can contribute to both attachment and transcytosis of HIV-1 through epithelia.

Altogether, regarding attachment and entry, these studies showed that HSPG serves a number of purposes during the early steps of HIV-1 dissemination, from capturing and presenting *in trans* free virions to replicative cells, to permitting the transfer of viral particles across epithelial or endothelial barriers (Figure 2). HSPG, when expressed by CD4^+^ permissive cells may also increase infectivity by favoring *in cis* viral particle concentration at the cell surface (see below).

A number of polyanions have been investigated for their ability to inhibit HIV-1 infection in clinical trials. In addition to suramin, dextran sulfate, and heparin, which were considered for systemic use, this includes many other sulfated/acidic compounds such as carrageenan, cellulose sulfate, polystyrene sulfonate or maleic acid, naphthalene sulfonate, and cellulose acetate phthalate, developed as anti HIV-1 microbicides. Unfortunately, none of these compounds proved to be effective *in vivo* presumably due to poor availability, toxicity, sequestration by plasma proteins, induced reduction in epithelial integrity and concomitant increases in permeability to HIV-1 particles (67, 68). Their variability, in terms of molecular weights and degrees of sulfation also made them difficult to standardize.

## The Cell Surface Ligands of gp120 and the Entry Process

### CD4, CCR5, and CXCR4

**Figure 3:**
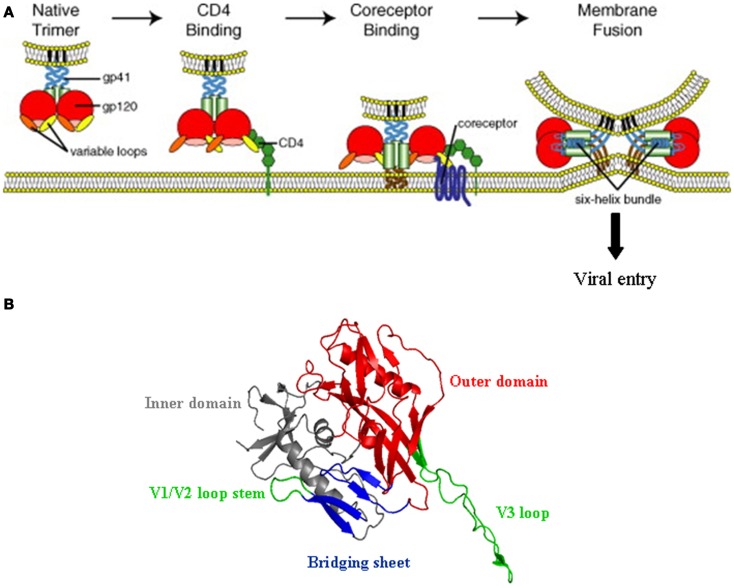
**HIV-1 entry mechanism**. **(A)** Schematic representation of the multi-step process of HIV-1 entry; from attachment to CD4 (left) to fusion between the viral and the cell membrane (right). The gp120 trimer, upon binding to CD4 (in green), experiences extensive structural changes that open up the variable loops V1/V2 and V3 (orange and yellow), and concomitantly expose and/or fold the so called CD4 induced bridging sheet that will be recognized by the co-receptor (CCR5 and/or CXCR4). This second interaction triggers the insertion of the gp41 fusion peptide into the cell membrane and promotes viral entry (Reprinted from Ref. (102), with permission from Elsevier). **(B)** Three-dimensional structure of gp120 in the CD4-bound conformation (from pdb:2b4c), showing the inner and outer domains, the V1/V2 loop stem, and the four β strands (CD4 induced bridging sheet in blue) that together with the V3 loop (in green) contribute to co-receptor selectivity and interaction.

Once in contact with permissive cells, i.e., cells that are CD4 and CCR5 and/or CXCR4 positive, the HIV-1 can start its replication cycle. HIV-1 entry into its target cells is initiated by a highly complex series of interactions, which first involve the binding of gp120 to its primary receptor, CD4 (69). This initial step not only enables the viral particles to attach to the cell, but also drives extensive structural alterations that primes the envelope for binding to either CCR5 or CXCR4 (70). This second interaction, which elicits further modifications in Env, triggers the activation of the gp41 fusion peptide whose insertion into the host membrane ultimately leads to the delivery of the viral contents into the host cytoplasm (Figure 3). The gp120 thus constitutes the central element for all interactive events occurring during the pre-entry steps and, accordingly, this molecule features several interactive regions and is structurally complex; It consists of five relatively conserved regions (C1–C5), that fold into a “core” structure organized into two distinct regions termed the “inner” and “outer” domains that are connected by a bridging segment, and five surface-exposed variable loops (V1–V5). The CD4 binding site is formed from conserved residues in discontinuous segments of the C1, C3, and C4 domains that are brought into proximity in the folded gp120 and located into a depression formed at the interface of the outer and inner domain and the region that connects these two domains (71). Binding to CD4 triggers extensive structural alterations, in particular within the inner domain of the protein. Although X-ray crystallographic analysis have led to atomic models for gp120 on which the V1/V2 and V3 loops were deleted, it seems likely that these structural modifications include both a relocation of the V3 and shifts of the V1/V2 loops, whose base in the inner domain (β2 and β3 strands) is brought into close proximity to a β-hairpin of the outer domain (β20 and β21 strands). This exposes new regions that, partially masked by the V1/V2 and V3 loops, were cryptic in the unliganded gp120, and concomitantly folds a four-stranded β-sheet located within the bridging sheet that connects the inner and the outer domain of the glycoprotein (71, 72). In conjunction with the V3 loop (73), this β-sheet (known as CD4i for CD4 induced epitope) makes up the binding site for either CCR5 or CXCR4 (70, 74, 75). The V3 loop has a major influence on HIV-1 tropism and appears to be a key determinant for co-receptor selectivity, which in turn, affects the overall process of viral pathogenesis. Its sequence is important for defining the extent to which the CD4-bound form of the gp120 interacts with CCR5 or CXCR4, and hence the ability of a particular HIV-1 virus to enter cells using either CCR5 (viruses called R5-tropic) or CXCR4 (X4-tropic viruses). R5X4 or dual tropic strains constitute a third class that can use either of these two co-receptors. It has been reported that in the majority of the infected subjects, the HIV-1 primarily uses CCR5 in order to initiate the infection. However, during the course of infection, the co-receptor usage preference of HIV-1 shifts from CCR5 to CXCR4 in 50% of the infected individuals, a change that is frequently associated with the accelerated CD4^+^ T-cell decline and the rapid progression toward AIDS. In the context of the interaction between gp120 and HS, it is worth noting that, in general, the R5 to X4 tropism switch is associated with an increase in the net positive charge of the V3 loop (75), which will also determine to which extent gp120 will interact with HS (see below).

### Heparan sulfate

Very early in the study of HIV-1, it was found that a number of HS like molecules, including heparin, dextran sulfate, and various heparinoids (such as pentosan polysulfate, fucoidan, curdlan sulfate) inhibit viral replication in cell culture experiments (76–78). This appeared to mostly occur by preventing HIV-1 binding to the cell surface as shown by the ability of heparinase treatment of HIV-1 sensitive lymphoblastic T-cell lines, such as MT-4 and H9, to reduce HIV-1 absorption to these cells (38, 79). Although some studies suggested that heparinoids could bind to CD4 and thus interfere with HIV-1 binding to its primary receptor, several investigations showed that both soluble heparin and cell surface HS interact with gp120 (see below) and target the V3 loop of the viral protein (80, 81). As this domain is not involved in CD4 binding, this excluded that heparinoid works by blocking the gp120-CD4 interaction, which was further confirmed by the observation that (i) HS could be immunoprecipitated from gp120 treated H9 cells with either anti gp120 or anti-CD4 antibodies (82), and (ii) dextran did not interfere with the binding of CD4 to recombinant gp120 at concentrations which effectively prevent HIV-1 replication (83, 84). Interestingly, it was shown that if the polyanionic nature of these compounds were essential for their *in vitro* anti-HIV-1 activities, a number of other sulfated polysaccharides, including for example various chondroitin sulfates (another member of the GAG family), have no such activities. This suggests that specific structural features of the polysaccharide might be important for activity. It thus appears that, in addition to CD4, cell surface HSPG functions as an attachment receptor recognized by the V3 loop of gp120, and therefore suggesting that this interaction allows the virus to scan the cell surface and could assist binding to specific entry receptors. Most of these studies however date back to the discovery of the HIV-1 co-receptors and their importance in tropism. They were performed with T-cell line-adapted HIV-1 that use CXCR4 to enter cells and immortalized T-cell lines that express large amounts of HSPG. The binding of HIV-1 to cell surface HSPG is however likely to depend on the level of expression of this molecule, and it was then reported that if cell surface HSPG facilitates HIV-1 entry into some cell lines it does not into primary T-lymphocytes (85, 86), questioning the physiological relevance of HSPG for capturing viral particles at the surface of CD4^+^ cells.

Primary T-lymphocytes and macrophages, the two major HIV-1 targets, feature opposite patterns of attachment receptors. In the former, which express high CD4 levels, chondroitin sulfate largely outnumbers HS moieties (87), while macrophages display low levels of CD4 but produce large amount of HS (88). On these cells, HIV-1 attachment is mostly mediated by HSPGs, and this interaction was found necessary for successful infection when a low level of CD4 is expressed at the cell surface (88, 89). Thus, HS may compensate for low level CD4 expression and induce a sufficiently high viral particle concentration for infection that CD4 by itself would not achieve. In contrast, the high levels of CD4 on T-lymphocytes obviate the need for other attachment molecules. Together, this shows that the role of HS in mediating *in cis* HIV-1 infection may depend on the cell surface CD4/HS ratio and their level of regulation. For example, it has been observed that while quiescent primary CD4^+^ T cells do not express detectable levels of HSPGs, HSPGs are expressed on primary CD4^+^ T cells following activation by interleukin-2/phytohemagglutinin or anti-CD3/anti-CD28 antibody. This immune activation coincides with binding and entry of HTLV-1, a known HS binding virus (90). Although determining the GAG nature/expression pattern and architecture of primary cells is not trivial, it might be of interest to better characterize the HS status of HIV-1 target cells during the evolution of the pathogenesis. Finally, it is also important to note that HIV-1 susceptibility to HS is dependant on the HIV-1 strain as all do not display high affinity for this GAG (see below).

## A Biochemical View on the gp120-HS Interaction

### The V3 loop is the major HS binding determinant

Early studies, investigating the mechanism by which polyanions such as heparin or dextran sulfate inhibit HIV-1 replication pointed out that these polysaccharides interact with the gp120 V3 loop. This was essentially based on the observation that polyanions block the binding of a number of monoclonal antibodies, directed against the V3 loop to either recombinant gp120 on ELISA plates or to HIV-1 infected cells expressing gp120 on their surface (80, 82, 84). These results were further confirmed with data showing that HS directly binds to V3 derived peptides (81, 91), whose sequences were characterized by Lys and Arg enriched clusters that are commonly found on protein HS binding sites. However, the V3 loop, a disulfide-bonded structure of approximately 35-residues-long is highly variable and prone to mutation-induced sequence changes (75). Its overall charge may vary from +2 to +10, with that of a CCR5-using HIV-1 strain generally in the range of +3 to +5 and that of a CXCR4-using isolate being from +7 to +10. Therefore, binding to HS may vary according to the gp120 origin and tropism, an evolution toward a more basic structure being linked to adaptation toward CXCR4 usage.

To investigate these points in more detail, the binding of heparin to WT and mutated forms of different gp120s, including MN (X4), HXBc2 (X4), 89.6 (R5X4), W61D (R5X4), Bal (R5), and JRFL (R5) were measured by different means: Surface Plasmon Resonance showed first that the X4-HXBc2 gp120 (whose V3 loop features nine positive charges) strongly binds to heparin, as does the R5X4-89.6 gp120 which has seven positive charges in its V3 loop. The binding of the W61D (another R5X4 gp120 with a V3 loop of +6) was reduced, and finally, the R5-gp120 (whose V3 loop contains only four basic residues) binds relatively weakly (gp120 Bal) or not at all (gp120 JRFL). These results were in agreement with another assay, in which the interaction of [^35^S]-labeled heparin to the above mentioned gp120s also showed that most of the variation in binding is due to changes in the charge and structure of the V3 loop. It was nevertheless observed that [^35^S]-labeled heparin binding to HXBc2 gp120 was not entirely suppressed by the V3 loop deletion, suggesting that other regions could contribute, albeit to a lesser extent. Binding of HXBc2 with more substantial deletions, performed to address which other regions of the X4-gp120 might be implicated, showed that deletion of the NH_2_- and COOH-termini and the V1/V2 loop structure resulted in a small loss of [^35^S]-heparin binding. An additional deletion of the V3 loop dramatically reduced [^35^S]-heparin binding and preincubation of this mutant with the monoclonal antibody 48d further reduced binding to background levels. These data suggest that whereas the V3 loop is the major determinant, the COOH- and NH_2_-termini, the V1/V2 loops and the bridging sheet between the inner and outer domains of gp120 (recognized by the 48d antibody) could contribute to some extent to the binding of polyanions. Consistently with the idea that the V3 loop is the primary high affinity binding site on gp120, at least for the X4 derived Env, molecular modeling of the electrostatic potential of the protein confirmed that the overall charge on the surface is dominated by the V3 loop (92).

The early use of polyanionic compounds such as dextran sulfate as anti- HIV-1 therapeutic agents has not been successful in clinical trials (93). One possible reason for the *in vivo* failure of the molecule (in addition to toxicity and poor bioavailability) could be related to the V3 loop charge differences between R5 and X4 viruses, the former being the phenotype associated with HIV-1 transmission and early infection, while the latter being the only one efficiently targeted by polyanions.

### HS binding to other surface exposed regions of gp120

To obtain unequivocal evidence for a direct interaction between polyanions and regions outside the V3 loops, a new approach designed to identify and simultaneously map potential HS binding sites on a protein surface was developed (94). This method, which uses unmodified native proteins, is based on the formation of cross-linked complexes of the protein of interest with heparin, followed by the proteolytic digestion of these complexes, and the subsequent identification of the heparin bound peptides by N-terminal sequencing using an automated protocol of Edman degradation and HPLC detection of the released amino acids.

Using this approach with HXBc2 gp120, three potential HS binding domains (HBDs) were consistently identified (including the V3 loop that was confirmed by this approach): RGKVQK (HBD 1: residues 166–171) located within the V2 loop, RKRIR (HBD 2: residues 304–308) at the base of the V3 loop and finally, KAKRR (HBD 3: residues 500–504), at the C-terminal domain of the protein (95).

Interestingly, all three HBDs are functionally important. HBD 1 and 2 in particular undergo important structural changes after CD4 binding, resulting in the unmasking of the co-receptor binding site and are thus key to the entry mechanism of the virus. As mentioned above, HBD 2 (within the V3 loop, which determines co-receptor usage) displays essential features for co-receptor binding. In particular, mutation of residues Arg298, Arg306, and Arg308, present in this HBD, strongly decrease the ability of gp120 to interact with CXCR4 (96). It has been reported that heparin enhances the furin cleavage of HIV-1 gp160 into gp120 and gp41, which occurs only three residues downstream HBD 3, but the significance of this observation is not clear (see Ref. (95) for discussion).

Together these data provide a direct demonstration of the existence of additional binding sites and identify some of the residues involved. They are consistent with a kinetic analysis of the gp120-heparin interaction which could be much better described by a complex model than by a single one-to-one binding mode (92) and with the observation that the V3 loop does not fully recapitulate the binding activity of the protein.

### A fourth HS binding site in gp120: The “CD4 induced” epitope

The gp120 molecules assume several distinct conformations and are characterized by an important intrinsic flexibility, which could likely influence heparin binding. As described above, and as shown by cryo-electron tomography on the native gp120 trimers (including the variable loops that were missing in the X-ray crystallographic analysis), the V1/V2 and V3 loops (comprising the HBD 1 and 2), in particular, are released and move away from the center of the Env spike (97) following binding to CD4. Thus, to further clarify the gp120-HS binding determinant, the unliganded monomeric gp120 (HXBc2 strain) and the gp120 in its CD4-bound conformation, were compared for their ability to interact with heparin. This showed that in its CD4-bound form, gp120 had a substantially increased affinity for the polyanion, suggesting that the CD4-bound conformation could have improved the accessibility of the V1/V2 and V3 loops and/or stabilize them into a structure better recognized by HS.

**Figure 4:**
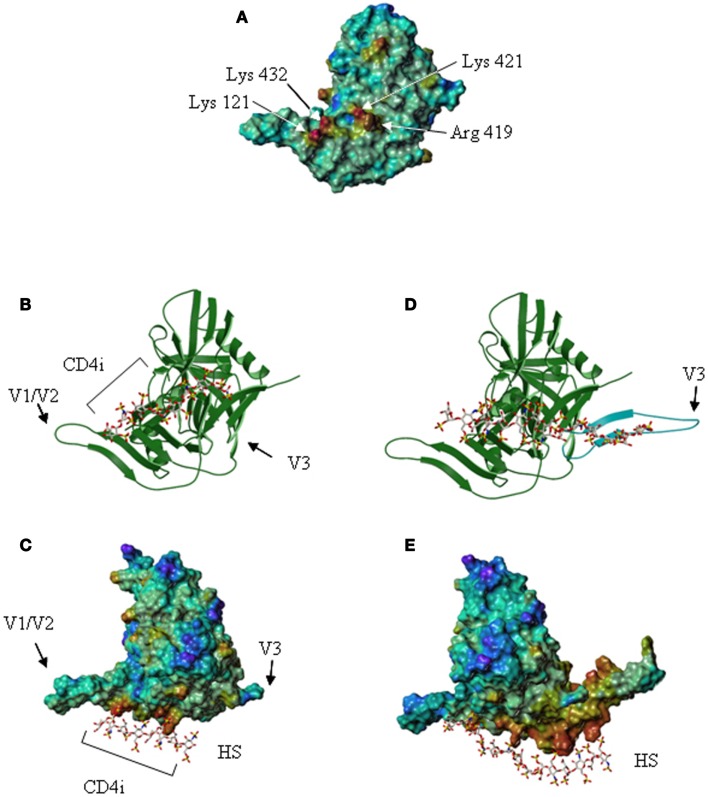
**The V3 loop and the co-receptor binding domain of gp120 features HS binding sites**. **(A)** The gp120 CD4-induced domain displays a HS binding structure. The Connolly surface of the HXBc2 gp120 core was color-coded according to the electrostatic potential from negative values (blue) to positive values (red). The basic residues of the CD4-induced (CD4i) epitope, which form a HS binding site, are indicated. **(B)** Representation of the lowest energy model of a gp120/HS derived octasaccharide complex. The protein [orientation as in **(A)**] is represented by a ribbon, and the octasaccharide by sticks. **(C)** Lowest energy model of the gp120 (on which the Connolly surface was calculated) in complex with a HS derived octasaccharide. V1/V2 and V3 indicate the stem of the V1/V2 and V3 loops. The location of the CD4i epitope is also indicated. **(D)** Structure of gp120, on which the V3 loop (in blue) was modeled. The HS binding residues of the V3 loop and the CD4-induced epitope are aligned on the surface of the protein and form an extended binding site on which has been docked a HS derived oligosaccharide of appropriate length. **(E)** Connolly surface of gp120, including the V3 loop, in complex with a tetradecasaccharide shown with the same orientation as in **(C)**.

However, CD4 binding also exposed the “CD4 induced” bridging sheet that was previously masked by the V1/V2 and V3 loops in the unliganded form. Examination of the gp120 electrostatic surface, using the structural data of the CD4-bound core glycoprotein, revealed that the CD4 induced region comprises a cluster of positively charged residues located between the stems of the V1/V2 and V3 loops, organized as a typical HS binding site (Figure 4). Using a molecular modeling approach to locate putative HS binding sites further shows that within this domain, amino acids Lys-121, Arg-419, Lys-421, and Lys-432 form a discontinuous surface with a linear shape extending up to 25 Å, which can be predicted by a GRID analysis to be the most favorable anchoring position for an oxygen atom from a sulfate group (98). These molecular modeling predictions could be confirmed by showing that HS and HS derived oligosaccharides strongly inhibited the binding of mAb 17b to the CD4-gp120 complex. The mAb 17b belongs to the CD4i antibody family and recognizes an epitope on the gp120 bridging sheet that is exposed upon CD4 binding. Interestingly, this sterically restricted region, which overlaps the binding site for the co-receptor, is a well conserved element amongst X4, R5, and dual tropic gp120. Mutagenesis then confirmed that of the four amino acids indicated above, Arg-419, Lys-421, and Lys-432 were key to the interaction with HS (95). All together, this defines an additional HS binding domain (HBD 4), located within the gp120 bridging sheet, importantly involved in co-receptor recognition and exposed only after CD4 binding.

## Development of a CD4-HS Glycoconjugate to Inhibit HIV-1 Attachment and Entry

**Figure 5:**
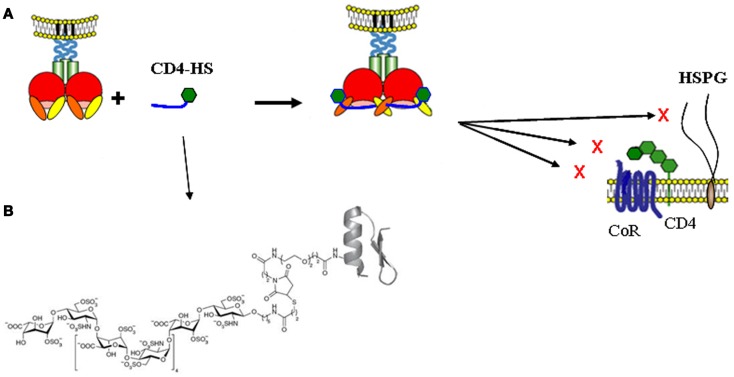
**Principe of inhibition of HIV-1 attachment and entry by “CD4-HS.”** **(A)** A CD4 mimetic peptide covalently linked to a HS dodecassaccharide (CD4-HS) bind to gp120 through its CD4 moiety and exposes the CD4i epitope, which then becomes available for interaction with the oligosaccharide. Such a bivalent molecule simultaneously binds to the CD4, the HS and the co-receptor binding sites of gp120 and blocks the interaction of the virus with all its principal cell surface ligands, inhibiting both attachment and entry (The gp120 was schematically represented as in Figure 3). **(B)** The structure of the mCD4-HS_12_ is also shown [modified from Ref. (99)].

These studies thus showed that HS binding to CXCR4 tropic gp120 is constitutive (the V3 loop -which dominates the interaction- is surface exposed), and can be enhanced by CD4 (which, together with V3 loop reorganization, exposes a new HS binding domain), while it is entirely CD4-induced for CCR5 tropic gp120. Interestingly, all these HBDs are located close to each other, at the proximity of- or within- the co-receptor binding site and are collectively involved in the conformational changes induced upon interaction with CD4 and in co-receptor recognition. In particular, mutations of Arg-419, Lys-421, Lys-432 within the bridging sheet, and Arg298, Arg306, Arg308, within the V3 loop, which are targeted by heparin, decrease the ability of gp120 to interact with CXCR4. This strongly suggested that polyanionic compounds, in addition to prevent HIV-1 association to cell surface HSPG, could directly block co-receptor binding and thus inhibit entry. However, being cryptic on the HIV-1 associated gp120, the co-receptor binding site needs to be exposed to be efficiently targeted. To this aim, a new molecule composed of CD4 covalently linked to HS has been prepared. To render this molecule potentially druggable it was based on a small CD4 mimetic (rather than recombinant CD4) and a chemically synthesized HS dodecamer (rather than natural derived HS, whose almost infinite structural variety would have made impossible the obtention of a defined compound). This chemically defined glycoconjugate (termed mCD4-HS_12_), whose size is 6000 Da, was shown to bind to gp120 through its mCD4 moiety and induce the structural modifications necessary to expose the co-receptor binding domain which therefore became available to be blocked by the HS_12_ moiety (Figure 5). This compound thus successfully targets two critical and highly conserved domains of gp120; the CD4 and the co-receptor binding domains. From a biochemical point of view, this compound blocks the binding of the prototypic R5-gp120 (YU2), to both CD4 and mAb 17b (directed against the co-receptor binding domain), while it blocks that of the prototypic X4-gp120 (MN) to CD4, HS and mAb 17b, with low nanomolar affinity (99). This molecule also prevents gp120 association to purified native CCR5 and CXCR4 co-receptors (100) and consequently displays a strong antiviral activity against R5- and X4- HIV-1 as well as against dual tropic virus with IC_50_ as low as 5 nM.

## Conclusion and Perspective

Investigation of the gp120 binding to HS has revealed a contrasting situation that is far more complex than previously thought. HIV-1 that uses CXCR4 as an entry co-receptor features up to four HBDs on their gp120, including the V1/V2 and V3 loops and the co-receptor binding site, while those using CCR5 mostly display HS binding activity essentially within the conserved and characteristically basic co-receptor binding domain. In that context, it is worth noting that the N-terminus of CCR5 and CXCR4 contain sulfotyrosine, as do a number of antibodies, directed against the gp120 co-receptor binding domains (101), also indicating that the gp120 CD4 induced region can be liganded by sulfated moieties.

Characterization of these HBDs, in particular within the CD4 induced surface, whose cryptic nature limits its accessibility both temporally and spatially during infection, led to the engineering of a new class of compounds in which HS was covalently linked to mCD4. These compounds, conceptually distinct from any other existing HIV-1 inhibitors, function by simultaneously exposing and blocking the HIV-1 co-receptor binding site, and therefore inhibit binding of gp120 to both CD4 and CCR5/CXCR4. It thus efficiently inhibits viral replication by blocking entry, which is currently considered as a compelling target for controlling viral replication (102). This molecule also inhibits the binding of gp120 to HS and has thus the potential for preventing viral adsorption on mucosa or viral transport through the blood brain barrier. It could therefore be further developed for both prevention and therapy following topical and/or parenteral application. In this regard, it is worth noting that these compounds are much more defined and have a much more specific mode of action than the above described polyanions that have been investigated up to now.

Despite tremendous progresses made in the development of antiviral drugs (103), HIV-1 continues to be a major health concern and remains one of the leading causes of death worldwide, which necessitate the development of new antivirals. With regards to inhibitor development, it is worth noting that mCD4-HS_12_ is bivalent. Multivalency has a number of functional advantages, such as achieving high affinity, and increasing strength and specificity for the binding site. It has been indeed found that the mCD4-HS_12_ is, by far, more active than either its moieties alone, each reciprocally enhancing the blocking activity of the other in a cooperative manner (99). Targeting the gp120 co-receptor binding site, which although is well conserved across various HIV-1 strains exists in a dynamic equilibrium between its unliganded- and CD4-bound conformations, might thus be relatively challenging. From a structural point of view, it is interesting that HS is characterized by considerable internal motion and variation in its local three-dimensional structure. The IdoA, in particular, also exists in a dynamic equilibrium between a chair and a twisted skew-boat form, which may itself represent the average of a rapidly fluctuating ensemble of related structures. The conformation of HS also depends on its local sequence, the presence of poorly sulfated GlcA-GlcNAc domains giving rise to chain flexibility (104). It is thus tempting to suggest that HS is well designed to interact with an ensemble of conformationally dynamic structures such as that of the co-receptor binding domain of gp120, the high specificity of the conjugated bivalent compound being brought by the mCD4 moiety.

Currently, the HS_12_ moiety of the molecule displays a regular and highly sulfated sequence, (2-*O*-sulfated iduronic acid linked to *N*- and 6-*O*-sulfated glucosamine), so that the exact sulfation pattern recognized by gp120 is hidden in the fully sulfated oligosaccharide. HS synthesis is notoriously difficult, and in view of its huge diversity (see Figure 1), the synthesis of a library addressing structure-activity relationships is not realistic. In an effort to pinpoint the sulfate groups that are functionally essential to gp120 binding, the HS was substituted with sulfotyrosine-containing HS mimetic peptide, the synthesis of which is much more straightforward, and more easily amenable to sequence-activity relationship investigation. This molecule compares very well with HS_12_, and when conjugated to mCD4 broadly inhibits the replication of several HIV-1 strains with an IC_50_ of 1 nM (100), thus, opening the route to future developments.

## Conflict of Interest Statement

The authors declare that the research was conducted in the absence of any commercial or financial relationships that could be construed as a potential conflict of interest.
